# Imperatorin ameliorates pulmonary fibrosis via GDF15 expression

**DOI:** 10.3389/fphar.2023.1292137

**Published:** 2023-12-04

**Authors:** Cheng-Fang Tsai, Yen-Chang Chen, Ya-Zhen Li, Chen-Teng Wu, Pei-Chun Chang, Wei-Lan Yeh

**Affiliations:** ^1^ Department of Medical Laboratory Science and Biotechnology, Asia University, Taichung, Taiwan; ^2^ Institute of New Drug Development, China Medical University, Taichung, Taiwan; ^3^ Department of Surgery, China Medical University Hospital, Taichung, Taiwan; ^4^ Department of Bioinformatics and Medical Engineering, Asia University, Taichung, Taiwan; ^5^ Department of Biochemistry, School of Medicine, China Medical University, Taichung, Taiwan

**Keywords:** imperatorin, growth differentiation factor 15, fibroblasts, lung, fibrosis

## Abstract

**Background:** Pulmonary fibrosis features in damaged pulmonary structure or over-produced extracellular matrix and impaired lung function, leading to respiratory failure and eventually death. Fibrotic lungs are characterized by the secretion of pro-fibrotic factors, transformation of fibroblasts to myofibroblasts, and accumulation of matrix proteins.

**Hypothesis/purpose:** Imperatorin shows anti-inflammatory effects on alveolar macrophages against acute lung injury. We attempt to evaluate the properties of imperatorin on the basis of fibroblasts.

**Methods:** In *in vitro*, zymosan was introduced to provoke pro-fibrotic responses in NIH/3T3 or MRC-5 pulmonary fibroblasts. Imperatorin was given for examining its effects against fibrosis. The mice were stimulated by bleomycin, and imperatorin was administered to evaluate the prophylactic potential *in vivo*.

**Results:** The upregulated expression of connective tissue growth factor (CTGF), α-smooth muscle actin (α-SMA), and collagen protein due to zymosan introduction was decreased by imperatorin in fibroblasts. Zymosan induced the activity of transglutaminase 2 (TGase2) and lysyl oxidase (LOX), which was also inhibited by the administration of imperatorin. Imperatorin alone enhanced sirtuin 1 (SIRT1) activity and growth differentiation factor 15 (GDF15) secretion in fibroblasts via LKB1/AMPK/CREB pathways. In addition, GDF15 exerted a beneficial effect by reducing the protein expression of CTGF, α-SMA, and collagen and the activities of TGase and LOX. Moreover, orally administered imperatorin showed prophylactic effects on bleomycin-induced pulmonary fibrosis in mice.

**Conclusion:** Imperatorin reduces fibrotic marker expression in fibroblasts and also increases GDF15 secretion via the LKB1/AMPK/CREB pathway, attenuating pro-fibrotic responses *in vitro*. Imperatorin also alleviates pulmonary fibrosis induced by bleomycin *in vivo*.

## 1 Introduction

Pulmonary fibrosis is a chronic lung disorder with limited therapeutic options. The excessive and continuous scarring of the lung can be idiopathic or secondary to various conditions. Damaged pulmonary structure and impaired lung function inevitably lead to respiratory failure and death. Regardless of the various mechanisms underlying initiation of fibrosis, it is commonly seen that pro-fibrotic cytokines and growth factors are increased locally or in the circulation during disease progression. Pro-fibrotic cytokines, such as transforming growth factor-β (TGF-β) and tumor necrosis factor-α (TNF-α), and growth factors, including connective tissue growth factor (CTGF), insulin-like growth factor 1 (IGF-1), and platelet-derived growth factor (PDGF), activate pulmonary fibroblasts (Luzina et al., 2015; Huaux, 2021; Phan et al., 2021; She et al., 2021; Effendi and Nagano, 2022). Activated fibroblasts transform into α-smooth muscle actin (α-SMA)-expressing myofibroblasts that deposit excessive extracellular matrix (ECM), such as collagen, leading to its accumulation, which characterizes fibrotic lungs (Horowitz and Thannickal, 2019).

Emerging evidence has revealed that ECM modification highly involves ECM cross-linkage and increased tissue stiffness. Transglutaminase 2 (TGase2) is an enzyme that cross-links glutamine and lysine residues of proteins and covalently modifies proteins, resulting in resistance to proteolysis, which consequently leads to matrix stability and tissue rigidity (Lorand and Graham, 2003). In addition, lysyl oxidase (LOX) oxidizes lysine and hydroxylysine residues in collagen to form covalent cross-linkage of collagen, thus making it insoluble in the ECM (Wordinger and Clark, 2014). On the other hand, sirtuin 1 (SIRT1) is an NAD^+^-dependent lysine deacetylase associated with cardiovascular and pulmonary diseases including fibrosis (Zerr et al., 2016; Liu et al., 2019; Mazumder et al., 2020). Growth differentiation factor 15 (GDF15), a divergent member of the TGF-β superfamily, is another factor that is involved in fibrosis (Kim et al., 2018a; Kim et al., 2018c). However, the role of GDF15 is paradoxically varied and depends on pathological conditions, target tissues, and downstream pathways.

As a naturally occurring furanocoumarin derivative, imperatorin has been studied for its pharmacological activities of anti-tumor, neuroprotection, anti-inflammation, and anti-hypertension (Nasser et al., 2019; Deng et al., 2020; Grabarska et al., 2020; Huang et al., 2021a; Huang et al., 2021b). In our previous study, we found that imperatorin is effective against pulmonary inflammation (Li et al., 2019). However, little is known about the effects of imperatorin against pulmonary fibrosis. Therefore, in the present study, we have demonstrated the beneficial effects of imperatorin *in vitro* and *in vivo*. The prominent potential and mechanism of imperatorin against pulmonary fibrosis manifests it as a promising candidate for drug development.

## 2 Materials and methods

### 2.1 Materials

The details of the chemical compounds and antibodies used are listed in Supplementary Figure S1.

### 2.2 Cell culture

The MRC-5 human lung fibroblast cell line was purchased from Thermo Fisher Scientific (Waltham, MA) and maintained in DMEM/F-12 medium supplemented with 10% fetal bovine serum (FBS) and 1% penicillin–streptomycin solution. The NIH/3T3 mouse fibroblast cell line was obtained from Bioresource Collection and Research Center (Hsinchu, Taiwan) and cultured in DMEM containing 10% calf serum and 1% penicillin–streptomycin solution. Both cell lines were cultured in 10-cm dishes and incubated at 37.5°C and 5% CO_2_.

### 2.3 Western blot analysis

The proteins extracted by RIPA buffer were quantified using the BCA Protein Assay Kit (Thermo Fisher Scientific, Waltham, MA). Equal amounts of proteins were subjected to SDS-PAGE. After transfer of proteins, PVDF membranes were blocked with 7.5% skim milk for 1 h followed by immunoblotting with primary antibodies at 4°C overnight. After a brief wash, the membranes were hybridized with a secondary antibody at room temperature for another 1 h. The blots were visualized using the Pierce™ ECL Western Blotting Substrate (Thermo Fisher Scientific, Waltham, MA) with Fuji X-ray films (Chen et al., 2021) or using the iBright FL1500 Imaging System with iBright Analysis software (Invitrogen, Waltham, MA). The signal intensity was quantitated using ImageJ (Schneider et al., 2012).

### 2.4 TGase2 activity assay

Cell lysates were harvested and subjected to TGase2 activity assay using TGase2 Activity Assay Kit in duplicate for each independent experiment. Standard procedures were conducted by following the manufacturer’s instructions (Novus, Centennial, CO).

### 2.5 LOX activity assay

Cell lysates were collected and subjected to LOX activity assay using Lysyl Oxidase Activity Assay Kit in duplicate for each independent experiment. The procedures were performed by following the standard protocol provided by the manufacturer (Abcam, Cambridge, United Kingdom).

### 2.6 SIRT1 activity assay

The cells were harvested under non-denaturing conditions and subjected to SIRT1 activity assay using SIRT1 Activity Assay Kit in duplicate for each independent experiment. The procedures were conducted by following the instructions provided by the manufacturer (Abcam, Cambridge, United Kingdom).

### 2.7 GDF15 enzyme-linked immunosorbent assay

Culture supernatants after the indicated treatment were collected and subjected to GDF15 enzyme-linked immunosorbent assay (GDF15 ELISA) in duplicate for each independent experiment. The procedures were performed by following the protocol provided by the manufacturer (Abcam, Cambridge, United Kingdom).

### 2.8 Bleomycin-induced pulmonary fibrosis in mice

Animal experiments were approved by the Institutional Animal Care and Use Committee (CMUIACUC-2021-164, China Medical University, Taichung, Taiwan). Male C57BL/6 mice aged 7–8 weeks were purchased from the National Laboratory Animal Center (Taipei, Taiwan) and randomly grouped. After performing anesthesia with 2% isoflurane, the mice were intratracheally administered with equal volumes (40 μL) of either bleomycin (2 mg/kg in saline) or saline within 15 s. After 1 h, imperatorin (2 or 4 mg/kg in 100 μL saline) was given once per day every other day through oral gavage for 21 days. The mice were sacrificed 24 h later from the last dosage, and bronchial alveolar lavage fluid (BALF) was collected using two 0.1 mL aliquots of sterile PBS. After harvesting BALF, the lungs were dissected and the superior lobes were taken and homogenized for hydroxyproline analysis, and the left lungs were taken and prepared for H&E staining and Masson’s trichrome staining.

For staining, the lung tissues were fixed with 10% formalin. After paraffin embedding, 5-micron-thick slices were cut and stained using H&E and trichrome staining kits.

For hydroxyproline analysis, lung homogenates were prepared, and the procedures were performed by following the instructions provided by the manufacturer (BioVision, Milpitas, CA) for measuring the hydroxyproline content in the lungs.

For GDF15 secretion, BALF was centrifuged at 1,200 g for 10 min, and the cell-free supernatant was used to measure the production of GDF15.

### 2.9 Statistics

The results were analyzed with one-way analysis of variance (ANOVA) and multiple comparison procedures (Holm–Sidak) using SigmaPlot software, and significance was defined as *p* <0.05. Values are expressed as mean ± SD of at least three independent experiments, unless stated otherwise.

## 3 Results

### 3.1 Imperatorin ameliorates zymosan-induced pro-fibrotic response in fibroblasts

**FIGURE 1:**
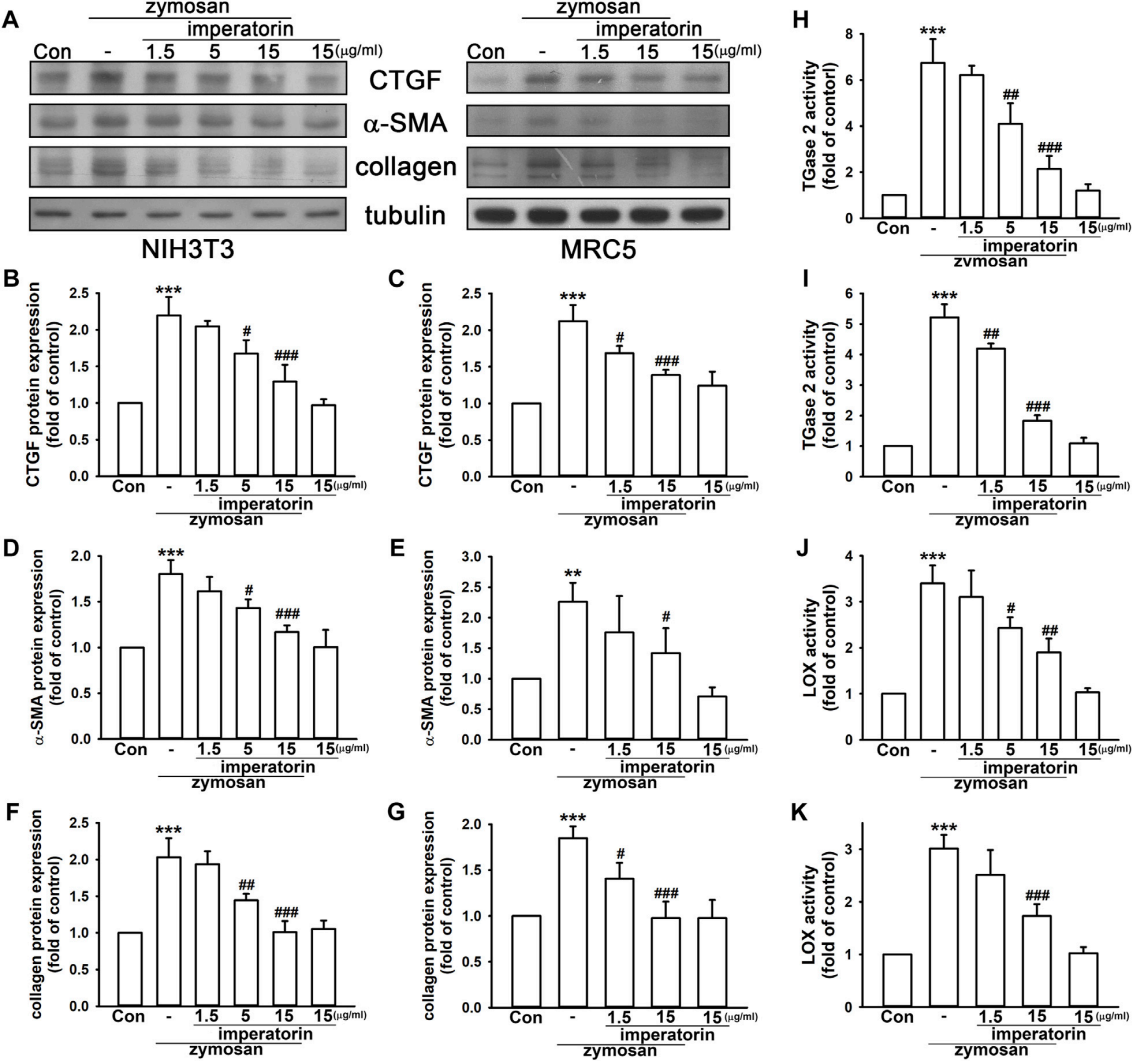
Imperatorin ameliorates zymosan-induced pro-fibrotic response in NIH/3T3 and MRC-5 fibroblasts. Representative images of protein expression are shown in **(A)**. Imperatorin (1.5, 5, and 15 μg/mL) was administered 30 min before zymosan (100 μg/mL) stimulation. After 24 h, the protein expression of CTGF, α-SMA, and collagen in NIH/3T3 **(B,D,F)** and MRC-5 fibroblasts **(C,E,G)** was examined by Western blotting. For measuring TGase2 and LOX activities, cell lysates were separately prepared after the same treatment in NIH/3T3 **(H,J)** and MRC-5 fibroblasts **(I,K)**. Note that imperatorin alone did not show significant effects on these pro-fibrotic markers. Graphs show the mean ± SD of three independent experiments. The *p*-value was calculated using one-way ANOVA and multiple comparison procedures (Holm–Sidak). ^**^ *p* <0.01, ^***^ *p* <0.001 compared to the control group. ^#^ *p* <0.05, ^##^ *p* < 0.01, ^###^ *p* <0.01 compared to the zymosan-treated group.

Our previous work (Li et al., 2019) showed that zymosan induces pulmonary inflammation that leads to immune cell infiltration and rapid lung fibrosis in mice. In this study, we have demonstrated that zymosan provoked the expression of fibrotic markers in MRC-5 and NIH/3T3 fibroblasts. As shown in Figures 1A–E, zymosan-induced CTGF production and α-SMA expression were significantly reversed by the pre-treatment of imperatorin in both MRC-5 and NIH/3T3 fibroblasts. Zymosan induced CTGF production by 2.20 ± 0.25- and 2.12 ± 0.22-fold in NIH/3T3 and MRC-5 fibroblasts compared to control cells, respectively, and the pretreatment of imperatorin followed by zymosan introduction reduced CTGF production by 1.29 ± 0.23- and 1.39 ± 0.07-fold compared to control cells, respectively. α-SMA was increased by zymosan by 1.80 ± 0.15- and 2.26 ± 0.31-fold in NIH/3T3 and MRC-5 fibroblasts compared to control cells, respectively, and 15 μg/mL imperatorin reduced zymosan-induced α-SMA expression by 1.17 ± 0.07- and 1.42 ± 0.41-fold compared to control cells, respectively. In addition, expression of α-SMA and collagen induced by 10 ng/mL of TGF-β were significantly abrogated by the treatment of imperatorin in MRC-5 fibroblasts (Supplementary Figure S2). Although CTGF was mildly elevated by TGF-β, imperatorin still caused the decreasing trend of CTGF expression.

Moreover, collagen accumulation and enzymes, including TGase2 and LOX, which stabilize matrix deposition, enhanced by zymosan were markedly reduced by the administration of imperatorin in a dose-dependent manner. As shown in Figures 1F–K, zymosan increased collagen expression by 2.03 ± 0.26- and 1.85 ± 0.13-fold in NIH/3T3 and MRC-5 fibroblasts compared to control cells, respectively, and 15 μg/mL imperatorin reduced zymosan-induced collagen by 1.01 ± 0.15- and 0.98 ± 0.18-fold compared to control cells, respectively. TGase2 activity was enhanced by zymosan by 6.75 ± 1.03- and 5.22 ± 0.43-fold in NIH/3T3 and MRC-5 fibroblasts compared to control cells, respectively, and the pretreatment of imperatorin with zymosan reduced TGase2 activity by only 2.13 ± 0.57- and 1.84 ± 0.18-fold compared to control cells, respectively. Similarly, LOX activity was enhanced by zymosan by 3.40 ± 0.38- and 3.01 ± 0.26-fold in NIH/3T3 and MRC-5 fibroblasts compared to control cells, respectively, and 15 μg/mL imperatorin inhibited zymosan-induced LOX activity by 1.90 ± 0.30- and 1.73 ± 0.23-fold compared to control cells, respectively. These data suggest that imperatorin is capable of inhibiting both zymosan- and TGF-β-induced pro-fibrotic responses by reducing the expression of fibrotic markers.

### 3.2 Imperatorin enhances SIRT1 activity and GDF15 secretion via the LKB1/AMPK/CREB signaling pathway in fibroblasts

**FIGURE 2:**
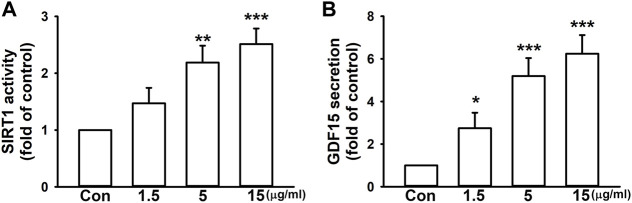
Imperatorin enhances SIRT1 activity and GDF15 secretion in NIH/3T3 fibroblasts. The cells were treated with imperatorin (1.5, 5, and 15 μg/mL) for 24 h. Cell lysates were harvested for examining SIRT1 activity **(A)**, and culture supernatants were collected for measuring GDF15 secretion **(B)**. Graphs show the mean ± SD of three independent experiments. The *p*-value was calculated using one-way ANOVA and multiple comparison procedures (Holm–Sidak). ^*^ *p* <0.05, ^**^ *p* < 0.01, ^***^ *p* <0.001 compared to the control group.

We surprisingly found that, without a pro-fibrotic stimulant, imperatorin alone enhanced SIRT1 activity by 2.51 ± 0.27-fold at the highest dosage in NIH/3T3 fibroblasts compared to control cells in a dose-dependent manner (Figure 2A). In addition, 15 μg/mL imperatorin also increased GDF15 secretion by 6.24 ± 0.88-fold in NIH/3T3 fibroblasts compared to control cells (Figure 2B).

**FIGURE 3:**
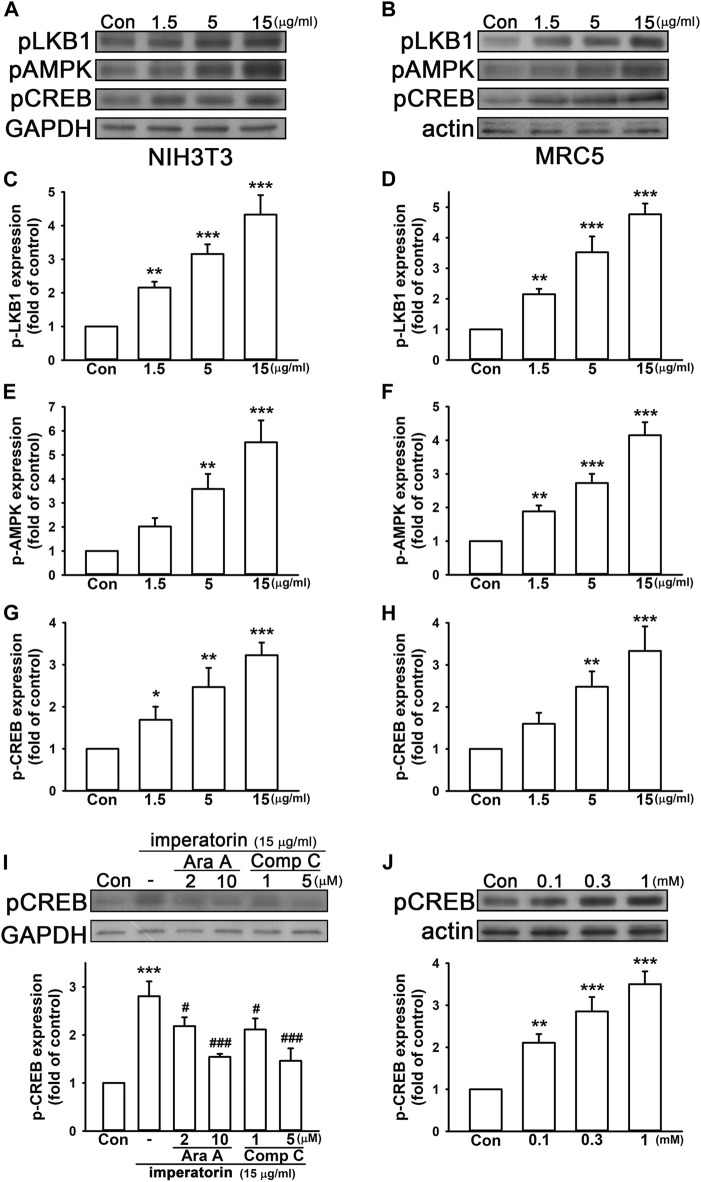
Imperatorin activates the phosphorylation cascade of LKB1/AMPK/CREB in NIH/3T3 and MRC-5 fibroblasts. The representative images of protein expression in NIH/3T3 **(A)** and MRC-5 fibroblasts **(B)** are shown. The cells were treated with imperatorin (1.5, 5, and 15 μg/mL) for 30 min, and the phosphorylation levels of LKB1, AMPK, and CREB in NIH/3T3 **(C,E,G)** and MRC-5 fibroblasts **(D,F,H)** were analyzed by Western blotting. **(I)** NIH/3T3 cells were pre-treated with ara-A (2 or 10 μM) or compound C (1 and 5 μM), which are both AMPK inhibitors, 30 min before imperatorin administration (15 μg/mL) for another 30 min. Note that imperatorin-induced CREB phosphorylation was antagonized by ara-A and compound C. **(J)** NIH/3T3 cells were treated with the AMPK activator AICAR (0.1, 0.3, and 1 mM) for 30 min, followed by the examination of CREB phosphorylation. Graphs show the mean ± SD of three independent experiments. The *p*-value was calculated using one-way ANOVA and multiple comparison procedures (Holm–Sidak). ^*^ *p* <0.05, ^**^ *p* <0.01, ^***^ *p* <0.001 compared to the control group. ^##^ *p* <0.01 compared to the imperatorin-treated group.

It has been reported that AMP-activated protein kinase (AMPK) and cyclic AMP (cAMP) response element-binding protein (CREB) are upstream of SIRT1 and may enhance SIRT1 activity (Noriega et al., 2011; Liu et al., 2021). Hence, we reasonably hypothesized that imperatorin may induce the phosphorylation level of AMPK and its upstream liver kinase B1 (LKB1) signaling pathways. As shown in Figure 3, 15 μg/mL imperatorin elevated the phosphorylation levels of LKB1, AMPK, and CREB by 4.33 ± 0.58-, 5.53 ± 0.91-, and 3.23 ± 0.30-fold in NIH/3T3 fibroblasts compared to control cells, respectively (Figures 3A, C; Figures 3E, G). Imperatorin also increased the expression of phospho-LKB1, phospho-AMPK, and phospho-CREB by 4.77 ± 0.35-, 4.15 ± 0.39-, and 3.33 ± 0.58-fold in MRC-5 fibroblasts compared to control cells, respectively (Figures 3B, D; Figures 3F, H). In order to exhibit the upstream and downstream cascade, AMPK inhibitors were used for examining whether phospho-CREB is affected. As shown in Figure 3I, inhibiting AMPK by ara-A or compound C in a dose-dependent manner antagonized imperatorin-induced CREB phosphorylation in NIH/3T3 fibroblasts. Furthermore, the activation of AMPK by AICAR markedly enhanced CREB phosphorylation by 3.50 ± 0.30-fold in a dose-dependent manner in NIH/3T3 fibroblasts compared to control cells (Figure 3J).

**FIGURE 4:**
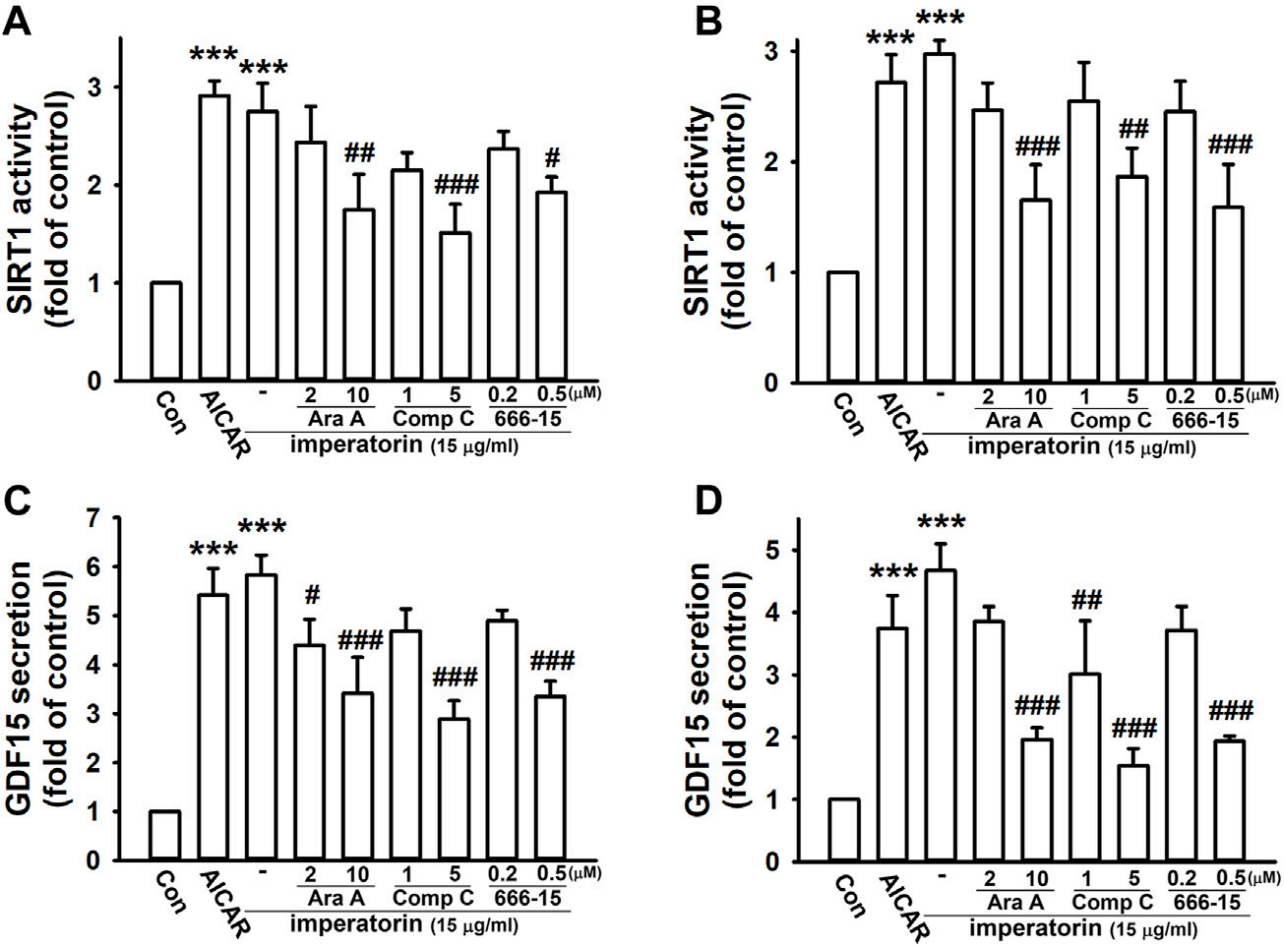
Imperatorin-induced SIRT1 activity and GDF15 secretion are mediated through AMPK/CREB in NIH/3T3 and MRC-5 fibroblasts. The cells were treated with the AMPK activator AICAR (1 mM) for 24 h or pre-treated with ara-A (2 or 10 μM), compound C (1 or 5 μM), or 666-15 (0.2 or 0.5 μM) for 30 min before imperatorin administration (15 μg/mL) for 24 h in NIH/3T3 **(A,C)** and MRC-5 fibroblasts **(B,D)**. Cell lysates were harvested for examining SIRT1 activity **(A,B)**, and culture supernatants were collected for measuring GDF15 secretion **(C,D)**. Graphs show the mean ± SD of three independent experiments. The *p*-value was calculated using one-way ANOVA and multiple comparison procedures (Holm–Sidak). ^***^ *p* <0.001 compared to the control group. ^#^ *p* <0.05, ^##^ *p* <0.01, ^###^ *p* <0.01 compared to the imperatorin-treated group.

To confirm that SIRT1 and GDF15 are regulated via the AMPK and CREB pathway, the pharmacological activators/inhibitors of AMPK and CREB were introduced. After activating AMPK by AICAR, SIRT1 activity and GDF15 secretion were significantly enhanced by 2.91 ± 0.15- and 5.42 ± 0.55-fold in NIH/3T3 fibroblasts, respectively, compared to control cells (Figures 4A, C). AICAR also increased SIRT1 activity and GDF15 secretion by 2.72 ± 0.25- and 3.74 ± 0.53-fold in MRC-5 fibroblasts, respectively, compared to control cells (Figures 4B, D). Inhibiting AMPK by ara-A and compound C or inhibiting CREB by 666-15 also noticeably abrogated imperatorin-induced SIRT1 activity and GDF15 secretion in both NIH/3T3 and MRC-5 fibroblasts. These results indicate that imperatorin-enhanced SIRT1 activity and GDF15 secretion are mediated by the LKB1/AMPK/CREB signaling cascade.

### 3.3 GDF15 exerts anti-fibrotic effects on fibroblasts

**FIGURE 5:**
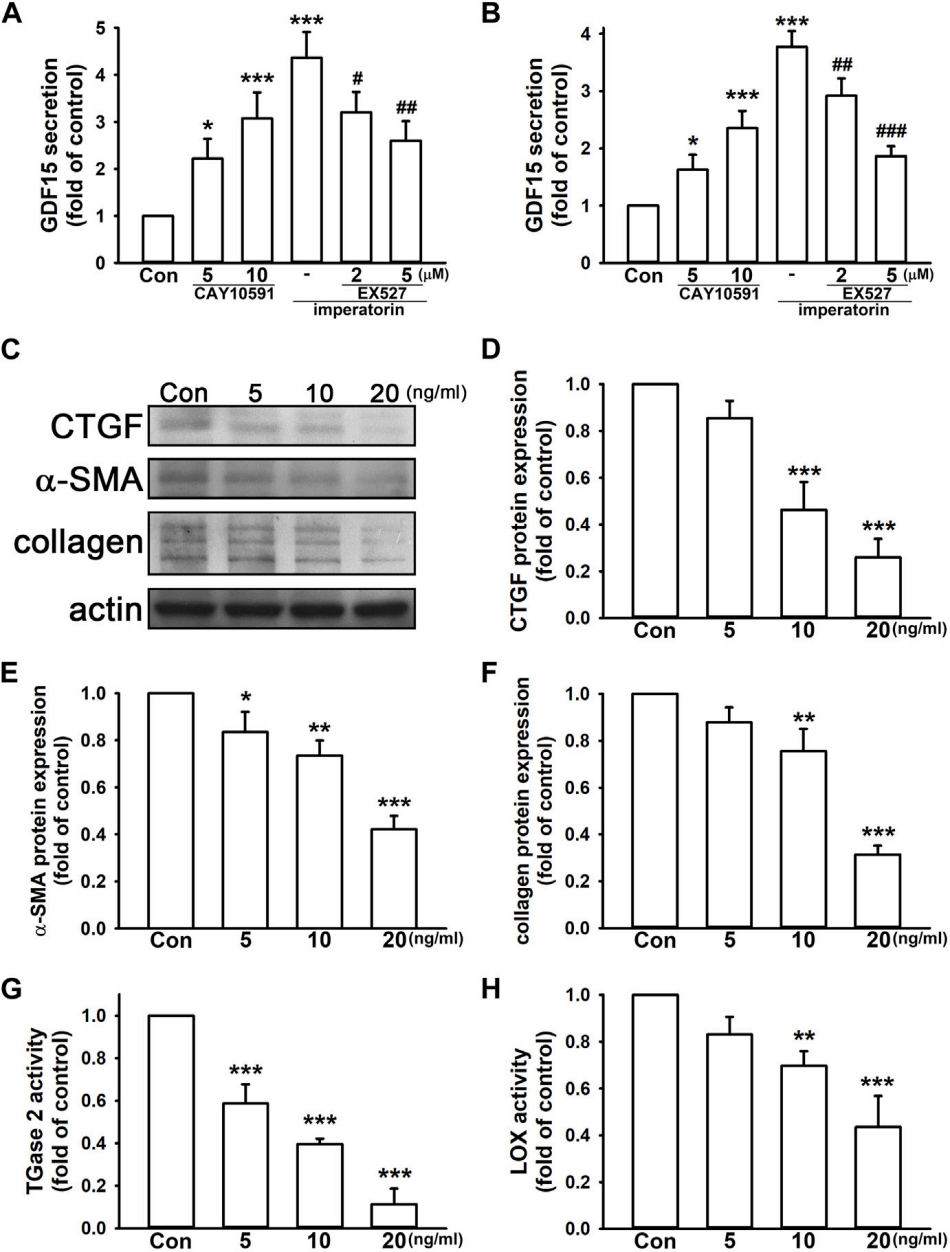
GDF15 exhibits anti-fibrotic effects in fibroblasts. The cells were treated with the SIRT1 activator CAY10591 (5 or 10 μM) for 24 h or pre-treated with the SIRT1 inhibitor EX527 (2 or 5 μM) for 30 min before imperatorin administration (15 μg/mL) for 24 h. Culture supernatants of NIH/3T3 **(A)** and MRC-5 fibroblasts **(B)** were collected for measuring GDF15 secretion. NIH/3T3 cells were treated with mouse recombinant GDF15 (5, 10, and 20 ng/mL) for 24 h, and the protein expression of CTGF, α-SMA, and collagen was examined by Western blotting **(D,E,F)**. Cell lysates were also subjected to TGase2 **(G)** and LOX activity **(H)** analyses. Representative images of protein expression are shown in **(C)**. Graphs show the mean ± SD of three independent experiments. The *p*-value was calculated using one-way ANOVA and multiple comparison procedures (Holm–Sidak). ^*^ *p* <0.05, ^**^ *p* <0.01, ^***^ *p* <0.001 compared to the control group. ^#^ *p* <0.05, ^##^ *p* <0.01, ^###^ *p* <0.01 compared to the imperatorin-treated group.

GDF15 has a controversy role in inflammation and fibrosis. We found that the SIRT1 activator, CAY10591, induced GDF15 secretion by 3.07 ± 0.56- and 2.35 ± 0.30-fold in NIH/3T3 and MRC-5 fibroblasts, respectively, compared to control cells in a dose-dependent manner (Figures 5A, B). Inhibition of SIRT1 by EX527 also reduced imperatorin-stimulated GDF15 secretion in a dose-dependent manner in both cell lines. As shown in Figures 5D, E, we found that the administration of GDF15 decreased CTGF and α-SMA expression by 0.26 ± 0.08- and 0.42 ± 0.06-fold in NIH/3T3 fibroblasts, respectively, compared to control cells. GDF15 also reduced collagen production by 0.31 ± 0.04-fold compared to control cells (Figure 5F). In addition, TGase2 and LOX activities were also inhibited by the administration of GDF15 by 0.11 ± 0.07- and 0.44 ± 0.13-fold, respectively, compared to control cells (Figures 5G, H). These findings indicate that GDF15 exhibits an anti-fibrotic effect on NIH/3T3 fibroblasts.

### 3.4 Imperatorin attenuates bleomycin-induced pulmonary fibrosis in mice

**FIGURE 6:**
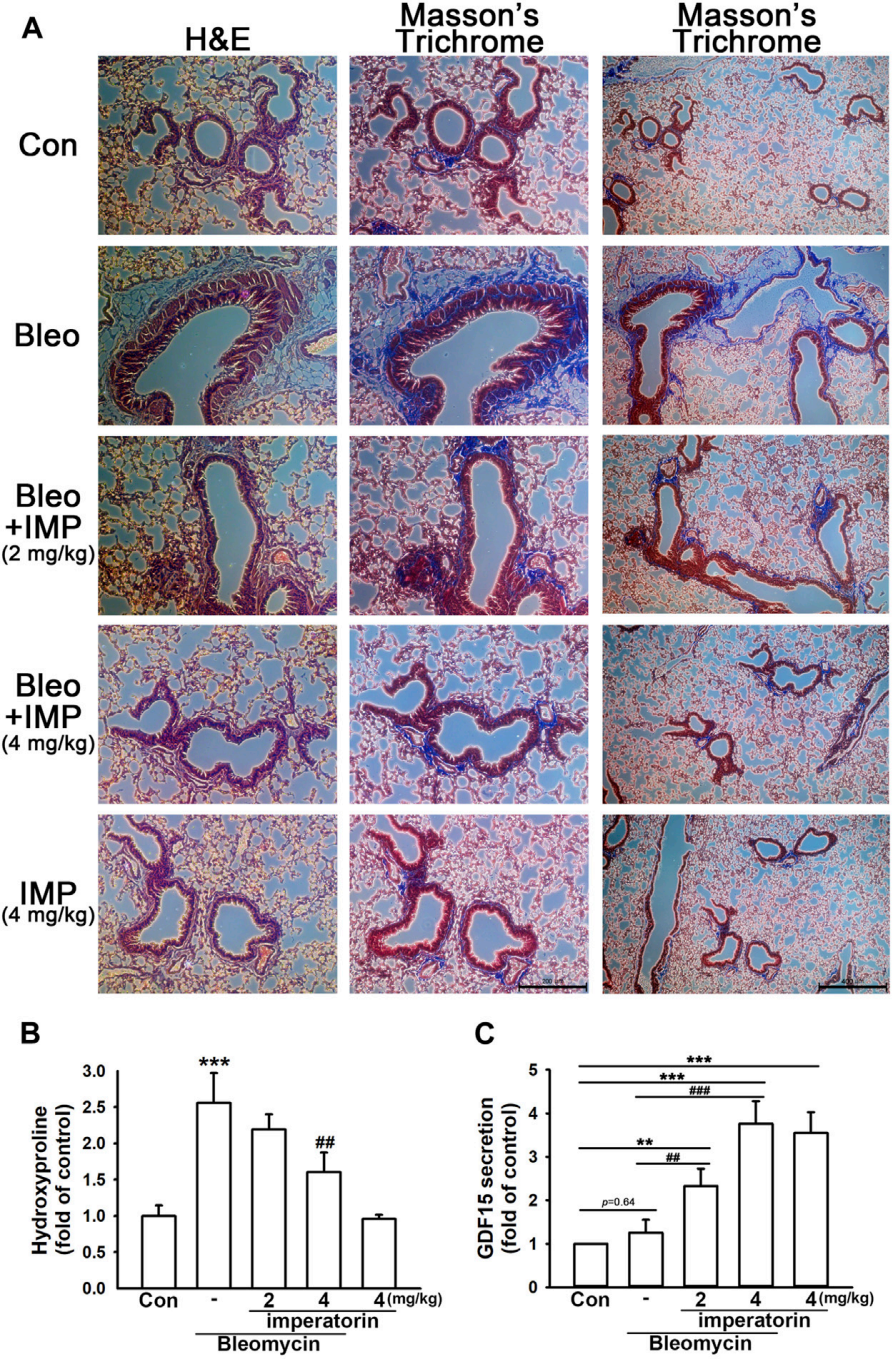
Imperatorin alleviates bleomycin-induced pulmonary fibrosis in mice. **(A)** Representative images of H&E and Masson’s trichrome staining of lung sections show that imperatorin (2 or 4 mg/kg) ameliorated bleomycin-induced pulmonary fibrosis in a dose-dependent manner. This fibrotic phenomenon was featured in basement membrane thickening, smooth muscle hypertrophy, and subepithelial collagen accumulation in the airway. There was also mild collagen deposition in the interstitium of bleomycin-stimulated mice. Hematoxylin stains the nuclei in blue, and eosin stains the cytoplasm in pink. Trichrome staining demonstrates collagen in blue, cytoplasm in pink, and nuclei in dark brown. The scale bar is 200 μm in the central panel and 400 μm in the right panel. Bleomycin-induced hydroxyproline production **(B)** was also decreased in a dose-dependent manner by orally administered imperatorin. **(C)** Expression of GDF15 in BALF was analyzed by GDF15 ELISA. Note that imperatorin enhanced GDF15 production regardless of the presence of bleomycin. Graphs show the mean ± SD of four independent experiments. The *p*-value was calculated using one-way ANOVA and multiple comparison procedures (Holm–Sidak). ^**^ *p* <0.01, ^***^ *p* <0.001 compared to the control group. ^##^ *p* <0.01, ^###^ *p* <0.001 compared to the bleomycin-treated group.

By performing H&E and Masson’s trichrome staining techniques, bleomycin-stimulated mice demonstrated significant basement membrane thickening, smooth muscle cell hypertrophy, and subepithelial collagen deposition in the airway. In addition, there was a visible collagen deposition in the interstitium; however, it was more significant in the airway. All of the aforementioned changes characterize pulmonary fibrosis (Figure 6A). With the administration of imperatorin, these appearances were attenuated both in the airway and interstitium in a dose-dependent manner. As shown in Figure 6B, the hydroxyproline assay also manifested that bleomycin-induced collagen deposition was alleviated by imperatorin administration, as bleomycin elevated hydroxyproline production by 2.58 ± 0.41-fold compared to control mice, and the co-administration of 4 mg/kg imperatorin reduced hydroxyproline production by only 1.60 ± 0.27-fold compared to control mice. As shown in Figure 6C, the secretion of GDF15 in BALF was analyzed by GDF15 ELISA. Imperatorin treatment significantly enhanced GDF15 secretion in a dose-dependent manner. With the co-treatment of bleomycin, 4 mg/kg imperatorin increased GDF15 expression by up to 3.76 ± 0.52-fold compared to control, while imperatorin alone increased GDF15 production by 3.55 ± 0.47-fold compared to control without bleomycin. These results indicate that imperatorin elevated GDF15 secretion regardless of bleomycin administration. Interestingly, bleomycin administration had slightly increased GDF15 production. This may be an internal protective mechanism that was carried out to defend pro-fibrotic stimulants. These results suggest that imperatorin is prophylactic against bleomycin-stimulated pulmonary fibrosis.

## 4 Discussion

There are different types of pulmonary fibrosis with different causes and histologies (Renzoni et al., 2021). Most of the pulmonary fibrosis types are associated with acute or chronic inflammation, accompanied by irreversible progressive scarring and thickening of tissues in the airway or pulmonary interstitium (Kreuter et al., 2021). Some pharmacological strategies propose novel molecules that tend to inhibit the inflammatory process, fibroblast differentiation, ECM component synthesis, and epithelial–mesenchymal transition and delay lung function decline and mortality, even without healing fibrosis (Savin et al., 2022). Currently, there are few FDA-approved treatments for pulmonary fibrosis. On the one hand, steroids are a class of medication that can treat certain types of pulmonary fibrosis by reducing inflammation. On the other hand, nintedanib (Richeldi et al., 2011; Richeldi et al., 2014; Flaherty et al., 2019) (a tyrosine kinase inhibitor) and pirfenidone (King et al., 2014; Behr et al., 2021) (an inhibitor of TGF-β, PDGF, and TNF-α singling) are anti-fibrotic therapies for idiopathic pulmonary fibrosis that can decelerate fibrosis and scarring. There is a clear need to develop useful alternative therapeutic strategies against pulmonary fibrosis. On top of our previous study showing that imperatorin exerts an anti-inflammatory effect on alveolar macrophages and alleviates rapid fibrotic injury *in vitro* and *in vivo* (Li et al., 2019), we aim at investigating the beneficial effect of imperatorin on fibroblasts and decelerating the establishment of pulmonary fibrosis *in vivo* in this study.

Evidence has suggested that AMPK and SIRT1 have similar effects on cellular metabolism, inflammation, and mitochondrial dysfunction by regulating each other and sharing common target molecules (Ruderman et al., 2010). Some reports suggest that AMPK is upstream of SIRT1 and can enhance SIRT1 activity (Canto et al., 2009; Liu et al., 2021), while others indicate that SIRT1 is required for AMPK phosphorylation and activation (Price et al., 2012; He et al., 2017). In addition, it has been identified that there are putative CREB-binding sites in the proximal promoter region of SIRT1, and it has been proven that SIRT1 promoter activity is induced by CREB; SIRT1 mRNA expression is also increased by the overexpression of CREB in HepG2 cells (Noriega et al., 2011). It has also been demonstrated that CREB directly mediates the transcription of SIRT1 by binding to SIRT1 chromatin and CREB deficiency markedly reduces the expression of SIRT1 (Fusco et al., 2012b). Moreover, SIRT1 and CREB may form a complex on the cAMP-responsive element, and SIRT1 may promote CREB transcriptional activity and the expression of target genes, creating a positive feedback loop (Fusco et al., 2012a; Fusco et al., 2012b). In this study, we have displayed that imperatorin enhances the phosphorylation of LKB1, AMPK, and CREB and subsequently elevates the activity of SIRT1. Inhibition of AMPK and CREB abolish imperatorin-induced SIRT1 activation and GDF15 expression.

This study is the first to reveal the protective effects of GDF15 that is regulated by SIRT1. Inversely different from our findings, SIRT1 is responsible for the protective effects of GDF15 by alleviating alveolar epithelial cell damage in a LPS-mediated acute lung injury model, and the beneficial effect of GDF15 is abrogated by SIRT1 suppression (Song et al., 2020). However, in this study, we demonstrated that GDF15 exerts the anti-fibrotic effect, and the expression of GDF15 is elevated downstream of SIRT1 activation. However, the exact mechanism underlying how SIRT1 mediates GDF15 expression requires further investigation.

Whether GDF15 is protective or harmful is controversial. GDF15 may even play divergent and paradoxical roles within the same pathological condition based on the tissues and downstream activated signaling, showing that GDF15 appears to have both beneficial and deleterious properties. GDF15 expression is elevated and is considered a marker of many pathological states, including inflammation, oxidative stress, and cancer (Han et al., 2008; Wang et al., 2013). While cigarette smoke extracts increase GDF15 production (Jiang et al., 2018), the concentrations of circulating GDF15 are generally increased in chronic obstructive pulmonary disease (COPD) compared with healthy individuals, where GDF15 contributes to mucin production in ciliated epithelial cells (Wu et al., 2012; Jiang et al., 2018), and the deletion of GDF15 leads to the amelioration of cigarette smoke-related pulmonary inflammation (Verhamme et al., 2017). In addition, GDF15 exhibits pro-fibrotic effects by activating fibroblasts and macrophages (Takenouchi et al., 2020). However, another study showed that GDF15 represses the TGF-Smad signaling pathway and leads to the inactivation of fibroblasts during pulmonary remodeling (Kim et al., 2018b). Furthermore, the GDF15 level positively indicates a poor prognosis in patients with acute respiratory distress syndrome (Clark et al., 2013; Kempf and Wollert, 2013). Nevertheless, it has also been demonstrated that GDF15 attenuates pulmonary inflammation induced by lipopolysaccharides (Song et al., 2020) or alleviates injury by reducing platelet–neutrophil aggregates (Herter et al., 2015). The anti-inflammatory effect of GDF15 is also exhibited in ischemia/reperfusion and septic injury (Kempf et al., 2006; Kempf et al., 2011; Abulizi et al., 2017). It is apparent that GDF15 does not exert a unifying character and its role is highly varied in different pathological states. In this study, GDF15 expression is induced by imperatorin, which in turn decreases the expression of pro-fibrotic markers, exerting a beneficial effect against pulmonary fibrosis.

Accumulating evidence demonstrates that pulmonary inflammatory damage occurs within 7 days after intratracheal stimulation, and second-stage fibrosis becomes gradually obvious on days 14–21 (Izbicki et al., 2002; Li et al., 2009). On the other hand, there are accumulating reports studying the beneficial effects and medicinal value of imperatorin. Despite the various pharmacological activities that have been reported, there are also a wide range of dosages of imperatorin that have been used. A measure of 5–50 mg/kg imperatorin shows anxiolytic effects and improves precognition activity (Budzynska et al., 2012). It has also been reported that 15–60 mg/kg imperatorin relieves allergic responses in mice (Xian et al., 2019). A measure of 50–100 mg/kg imperatorin inhibits cancer cell proliferation in mice (Mi et al., 2017). In our previous study, 4 mg/kg imperatorin protects mice from zymosan-induced pulmonary inflammation and rapid fibrosis (Li et al., 2019). In addition, high imperatorin dosages may cause extended prothrombin time and symptoms of poisoning, such as a loss of appetite, hepatocellular necrosis, and hypotension in rats (Awaad et al., 2012; Gong et al., 2014). Kidney damage and liver damage are also observed in mice (Gong et al., 2014). Taking into account the toxicity and side effects that may occur, we used imperatorin within a low-dosage range. Herein, we have demonstrated the beneficial effects of imperatorin in bleomycin-induced mice at a dosage of 4 mg/kg.

It is well known that during pulmonary fibrosis, there are several different cell types, such as epithelial cells, macrophages, and fibroblasts, orchestrating the fibrotic process from the early phase to the later phase. Evidence from our previous study shows that imperatorin exerts anti-inflammatory properties under pulmonary inflammatory conditions in alveolar macrophages and *in vivo* (Li et al., 2019). Here, in this study, we further confirm that imperatorin reduces fibrotic responses in fibroblast cell lines, indicating that imperatorin, taken together, may exhibit multiple beneficial effects on different cell types during the progression of pulmonary fibrosis.

In summary, this study has demonstrated the prophylactic effect of imperatorin against zymosan-induced pro-fibrotic responses by reducing the expression of CTGF, α-SMA, and collagen and the activities of TGase2 and LOX. Furthermore, imperatorin alone can increase SIRT1 activity via the LKB1/AMPK/CREB pathway, which mediates GDF15 secretion that reduces the pro-fibrotic responses. Moreover, evidence suggests that imperatorin alleviates pulmonary fibrosis *in vivo*, indicating the beneficial property of imperatorin against pulmonary fibrotic diseases.

## Data Availability

The original contributions presented in the study are included in the article/Supplementary Material; further inquiries can be directed to the corresponding author.

## Abbreviations

AMPK, AMP-activated protein kinase; BALF, bronchial alveolar lavage fluid; COPD, chronic obstructive pulmonary disease; CREB, cyclic AMP (cAMP) response element-binding protein; CTGF, connective tissue growth factor; ECM, extracellular matrix; GDF15, growth differentiation factor 15; LKB1, liver kinase B1; LOX, lysyl oxidase; α-SMA, α-smooth muscle actin; TGase2, transglutaminase 2; TGF-β, transforming growth factor-β; TNF-α, tumor necrosis factor-α; SIRT1, sirtuin 1.
