# Supplementary material for: Imperatorin ameliorates pulmonary fibrosis via GDF15 expression
Source: Front Pharmacol. 2023 Dec 4;14:1292137. doi: 10.3389/fphar.2023.1292137 (PMC10725920; doi:10.3389/fphar.2023.1292137)
Supplement: Supplementary file 1 [file DataSheet1.pdf]

| Antibody | Cat. No | Brand |
|---|---|---|
| α-SMA | ab5694 | Abcam (Cambridge, UK) |
| α-tubulin | T5168 | Sigma-Aldrich (St. Louise, MO) |
| β-actin | A5441 | Sigma-Aldrich (St. Louise, MO) |
| Collagen type I | 600-403-103 | Rockland (Limerick, PA) |
| CTGF | ab6992 | Abcam (Cambridge, UK) |
| GAPDH | G8795 | Sigma-Aldrich (St. Louise, MO) |
| p-LKB1 | 3482 | Cell Signaling (Danvers, MA) |
| p-AMPK | 2535 | Cell Signaling (Danvers, MA) |
| p-CREB | SC-81486 | Santa Cruz (Santa Cruz, CA) |
| Anti-mouse IgG-HRP | SC-2005 | Santa Cruz (Santa Cruz, CA) |
| Anti-rabbit IgG-HRP | SC-2004 | Santa Cruz (Santa Cruz, CA) |

| | | |
|---|---|---|
| GDF15 ELISA (Mouse) | Ab216947 | Abcam (Cambridge, UK) |
| GDF15 ELISA (Human) | Ab155432 | Abcam (Cambridge, UK) |
| Hydroxyproline assay kit | Abx298833 | Abbexa (Cambridge, UK) |
| LOX activity assay kit | Ab112139 | Abcam (Cambridge, UK) |
| SIRT1 activity assay kit | ab156065 | Abcam (Cambridge, UK) |
| TGase 2 assay kit | NBP1-37008 | Novus (Centennial, CO) |

| Chemical | Cat. No | Brand |
|---|---|---|
| AICAR | SC-200659A | Santa Cruz (Santa Cruz, CA) |
| Ara-A | CDS020711 | Sigma-Aldrich (St. Louise, MO) |
| Bleomycin | Ab142977 | Abcam (Cambridge, UK) |
| CAY10591 | 839699-72-8 | Cayman Chemicals (Ann Arbor, MI) |
| Compound C | 171260 | Sigma-Aldrich (St. Louise, MO) |
| EX527 | 49843-98-3 | Cayman Chemicals (Ann Arbor, MI) |
| Imperatorin | 482-44-0 | ChemFaces (Hubei, People Republic of China) |
| Recombinant mouse GDF15 | NBP2-51921 | Novus (Centennial, CO) |
| Recombinant human TGFβ | 100-21 | Peprotech (Cranbury, NJ) |
| Zymosan | Z4250 | Sigma-Aldrich (St. Louise, MO) |
| 666-15 | 538341 | Sigma-Aldrich (St. Louise, MO) |

**Supplementary Figure 1. Antibodies and chemicals used in this study.**

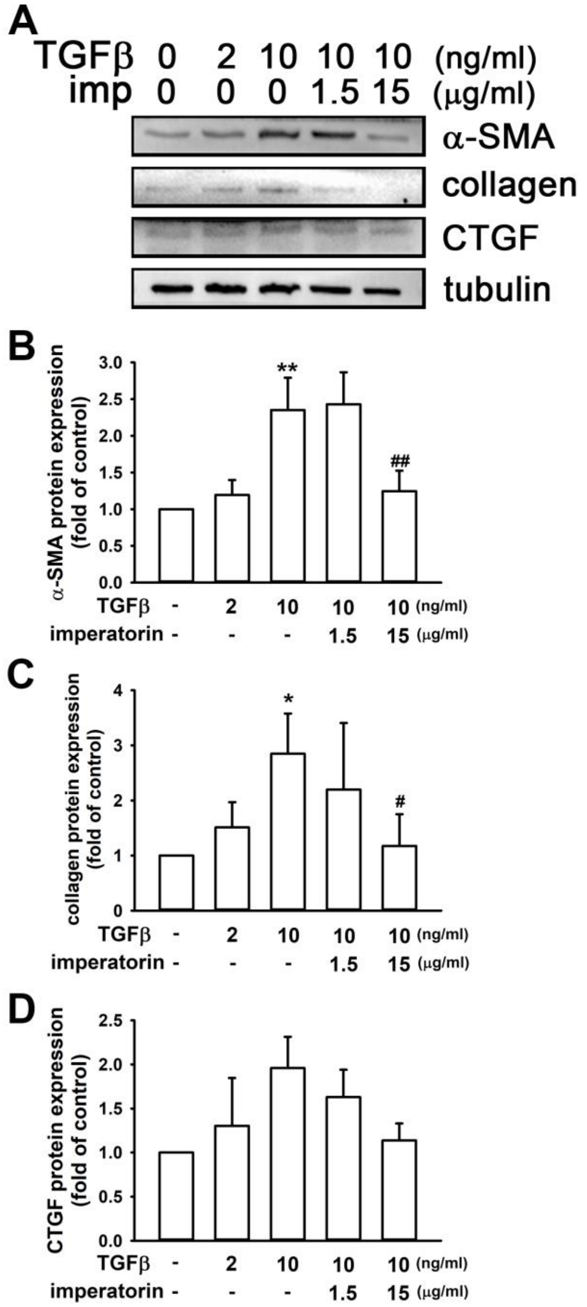


**Supplementary Figure 2. Imperatorin reduces TGFβ-induced pro-fibrotic response in MRC-5 fibroblasts.**

Representative images of protein expressions are shown in (**A**). Imperatorin (imp, 1.5 or 15 µg/ml) was administered 30 minutes before TGFβ (2 or 10 ng/ml) stimulation. 24 hours later, protein expressions of α-SMA (**B**), collagen (**C**), and CTGF (**D**) in MRC-5 fibroblasts were examined by Western Blotting. Graphs showed mean ± S.D. of three independent experiments. *p* value was calculated using one-way ANOVA and multiple comparison procedures (Holm-Sidak). $^{*}p < 0.0$; $^{**}p < 0.01$ compared to control group. $^{\#}p < 0.05$; $^{\#\#}p < 0.01$ compared to TGFβ-treated group.
